# The structures of natively assembled clathrin-coated vesicles

**DOI:** 10.1126/sciadv.aba8397

**Published:** 2020-07-22

**Authors:** Mohammadreza Paraan, Joshua Mendez, Savanna Sharum, Danielle Kurtin, Huan He, Scott M. Stagg

**Affiliations:** 1 Institute of Molecular Biophysics, Florida State University, 91 Chieftan Way, Tallahassee, FL 32306, USA.; 2 Department of Physics, Florida State University, 77 Chieftan Way, Tallahassee, FL 32306, USA.; 3 Department of Chemistry and Biochemistry, Florida State University, 95 Chieftain Way, Tallahassee, FL 32306, USA.

## Abstract

Clathrin-coated vesicles mediate trafficking of proteins and nutrients in the cell and between organelles. Proteins included in the clathrin-coated vesicles (CCVs) category include clathrin heavy chain (CHC), clathrin light chain (CLC), and a variety of adaptor protein complexes. Much is known about the structures of the individual CCV components, but data are lacking about the structures of the fully assembled complexes together with membrane and in complex with cargo. Here, we determined the structures of natively assembled CCVs in a variety of geometries. We show that the adaptor β2 appendages crosslink adjacent CHC β-propellers and that the appendage densities are enriched in CCV hexagonal faces. We resolve how adaptor protein 2 and other associated factors in hexagonal faces form an assembly hub with an extensive web of interactions between neighboring β-propellers and propose a structural model that explains how adaptor binding can direct the formation of pentagonal and hexagonal faces.

## Teaser

Distribution of adaptor proteins in clathrin-coated vesicles is not random.

## INTRODUCTION

Clathrin-mediated vesicle trafficking in eukaryotes is involved in most endocytic pathways (*1*), trafficking from trans-Golgi network (*2*), trafficking from endosomes (*3*), and also in the critical function of regenerating synaptic vesicles (SVs) (*4*–*6*). Clathrin and its associated adaptor/accessory proteins together carry out three important functions, which include engaging with cargo and membrane and also generating negative curvature on the membrane such that the cargo becomes encapsulated in a vesicle. Each of these functions is carried out by multiple adaptor/accessory proteins (*7*–*9*). Clathrin plays a major role in facilitating the formation of spherical vesicles (*10*–*12*) by making closed fullerene-like cages of different sizes both in vitro and in vivo (*13*, *14*). Clathrin together with the adaptors adaptor protein 1 (AP1) and AP2 act as assembly hubs because of their multiple binding sites for other adaptor/accessory proteins such as AP180 and epsin (*15*–*17*). The dynamic assembly of clathrin-coated vesicles (CCVs) has been investigated by super-resolution time-resolved fluorescence microscopy (*8*, *11*, *18*, *19*), and the self-assembly properties of clathrin have been understood by cryo–electron microscopy (cryo-EM) (*14*, *20*, *21*). However, it remains to be explained how clathrin assembly progresses into a closed cage with tight restraints on the final configuration of hexagonal and pentagonal faces (*22*) in a mechanism that primarily serves to transport cargo in lipid vesicles irrespective of cage geometry.

Clathrin is a 190-kDa protein with an N-terminal WD40 β-propeller domain (residues 1 to 330) followed by three helical zigzag domains in the shape of a leg and a C-terminal trimerization domain that assembles a clathrin triskelion (fig. S1, A and B), which is, in turn, the basic assembly unit of the clathrin cage. Clathrin alone can self-assemble into cages in vitro in acidic buffer because of the formation of multiple salt bridges between the heavy chains. Clathrin light chain (CLC), on the other hand, has a regulatory role. In physiological pH, CLC has been shown to inhibit cage assembly via its N-terminal EED motif (*21*, *23*). This inhibition was proposed to occur because of the interaction between the EED motif and the clathrin heavy chain (CHC) knee domain residues (R_1161_KKAR_1165_). For proper assembly, CHC’s knee domain can be bent when the interaction with CLC is absent. CLC’s inhibition on assembly is removed in vivo by Huntingtin interacting protein 1 (HIP1) (*24*), which interacts with the light chain N-terminal conserved sequence (residues 20 to 41) via its DLLRKN-containing coiled-coil domain. In addition, CLC also has a central heptad repeat and a C-terminal helical domain that interact with the CHC proximal domain and the trimerization helices, respectively. It has been shown through fluorescence resonance energy transfer measurements that the light chain heptad repeat undergoes a conformational change upon cage assembly. With these interactions, CLC is thought to make CHC cages more rigid, which is supported by direct measurements of the stiffness of the clathrin lattice using atomic force microscopy (*25*).

Numerous adaptor proteins link the clathrin cage to the membrane and cargo. AP2 as well as AP1, AP3, AP180, HIP1, and epsin have multiple linear motifs (clathrin boxes) in their C-terminal unstructured regions that bind the grooves of the CHC N-terminal β-propeller domain (*26*). AP2 is a heterotetrameric complex that is involved primarily in endocytosis and is composed of two large subunits, the α2 and β2 adaptins, and two small subunits, μ2 and σ2 adaptins, whose N-terminal domains form the core complex. The α2 adaptin mediates binding to membrane mainly through interactions with phosphatidylinositol 4,5-bisphosphate [PtdIns(4,5)*P*_2_]. The α2 and β2 adaptins have C-terminal clathrin box motifs and appendage domains that have been shown to bind clathrin (*16*, *27*, *28*). Although clathrin box–binding sites are well known, the appendage-binding site has been mapped to a broad N-terminal region of CHC including the β-propeller domain and helical zigzags (*29*). With its ability to recruit clathrin, phosphatidylinositol 4,5-bisphosphate [PI(4,5)P_2_], cargo recognition peptides, and other adaptor/accessory proteins, AP2 (and its counterpart AP1 in Golgi/endosome trafficking) serves as a nucleator of CCV assembly. However, it has remained unclear how AP2 can structurally organize the formation of closed clathrin cages in vivo.

Clathrin triskelia assemble into cages with pentagonal, hexagonal, and rarely heptagonal faces. There are 12 pentagons in a cage with the exception of two cage geometries, which contain heptagonal faces. As with any fullerene-like structure, curvature is only generated by the presence of pentagonal faces and the diameter dictated by the number of hexagonal faces. In a fully assembled clathrin cage, the interactions that associate CHC chains occur extensively at the edges, where four different CHCs interact along their proximal and distal domains. A 4.7-Å structure of clathrin cages was published recently that revealed that those interactions are highly conserved regardless of the cage size or face geometry (*14*). In that study, structures were determined of in vitro assembled cages together with the β2 appendage domain of AP2, but notably, they did not observe the β2 appendage density. However, in vivo, clathrin chains are brought together by the adaptor recruitment of clathrin at the N-terminal domain, which is positioned far from the edges. Therefore, it remains to be understood how adaptor-clathrin interactions are orchestrated with clathrin-clathrin interactions.

Here, using cryo-EM, we have determined the structures of natively assembled CCVs that include clathrin, adaptors, accessory proteins, and cargo. Our structures show a number of previously unresolved features including AP2 β2 appendages that cross-link adjacent CHC β-propellers. The appendage densities are heavily enriched in hexagonal faces, and by performing a focused alignment and classification, we resolve how AP2 and other associated factors in hexagonal faces form an assembly hub, resulting in an extensive web of interactions between neighboring β-propellers. In addition, we resolved a clathrin geometry that was previously predicted to not exist because of unfavorable bent conformations in some of the faces. Together, these observations led us to propose a structural model that explains how adaptor binding can direct the formation of pentagonal and hexagonal faces.

## RESULTS

### Natively assembled CCVs assume five different cage geometries including one not thought to be possible

CCVs were purified from bovine brains in conditions that ensured that CHC cages do not disassemble during purification. SDS–polyacrylamide gel electrophoresis gels showed bands that corresponded to CHC and CLC and adaptor subunits (fig. S2A). The identities of these proteins and other accessory and cargo proteins were determined by mass spectrometry (fig. S2A). This showed that the adaptors AP2 and AP180 were highly abundant followed by AP1 and AP3. Other essential accessory proteins were present in the CCVs, including AP2-associated kinase 1, epsin, HIP1, and amphiphysin. Besides AP2 and AP180, all the other essential components of SVs (*30*) were present in the spectra, including the following: vesicle-associated protein 2 (VAMP2) [R–soluble *N*-ethylmaleimide–sensitive factor attachment protein receptor (R-SNARE protein)], synaptophysin (VAMP2 chaperone and inhibitor), synapsin (localizes SVs to the synapse pool), sodium-potassium adenosine triphosphatase (ATPase) pump and H^+^-dependent ATPase pump, and neurotransmitter transporters (fig. S2A). SV-related proteins had the highest number of unique peptides and total normalized spectra in the mass spectrometry analysis, reflecting their higher concentration in the purified CCVs, and are consistent with the SV protein composition that was identified by Takamori *et al.* (*31*). Other identified proteins include syntaxin 1 and 2 (plasma membrane), syntaxin 7 (early endosome, late endosome), and syntaxin 16 (trans-Golgi network).

**Fig. 1:**
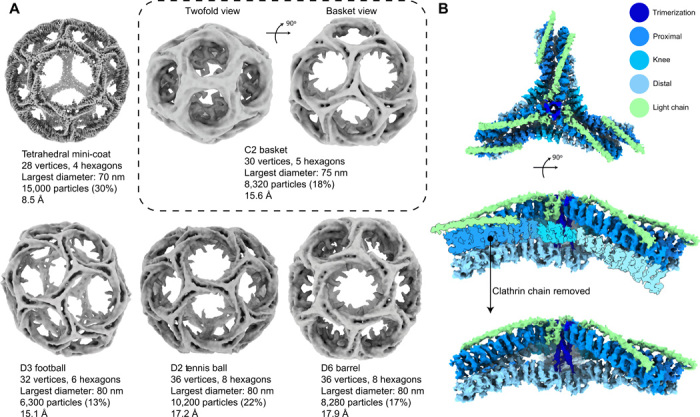
Single-particle refinement of CCVs. (**A**) Refinement of CCVs into five different cage geometries. The tetrahedral mini-coat has the highest resolution among the geometries. The C2 basket is a previously uncharacterized geometry represented in two views in the dashed panel. The largest cages are the D6 barrel and the tennis ball. (**B**) Clathrin-focused subparticle refinement of the mini-coat asymmetric vertex further improves the resolution. Top: Asymmetric vertex refinement with colors representing different domains; resolution, 6.3 Å (FSC_0.143_). Middle: Side view of the vertex. Bottom: Side view with one CHC and CLC removed to show the trimerization helices.

The natively assembled CCVs were prepared for cryo-EM, and a single-particle cryo-EM dataset was collected, resulting in 80,000 CCV particles (fig. S2B). These were subjected to Relion’s ab initio model generation (*32*) with a target of four models. This resulted in four CCV structures with complete cages and unique symmetries, including the mini-coat (tetrahedral, 28 triskelia, EMD-21608), football [D3, 32 triskelia, EMD-21612, also known as sweet potato (*14*)], tennis ball (D2, 36 triskelia, EMD-21613), and D6 barrel (D6, 36 triskelia, EMD-21614) (Fig. 1A and fig. S3A). However, after inspection of the individual particles, it was clear that there were more geometries present in the dataset. Therefore, the cages were subjected to multiple rounds of competitive three-dimensional (3D) classification, where the particles that were already assigned to one class were further classified. This led to the identification of a subset of particles that form a previously undescribed geometry with C2 symmetry and 30 triskelia that we are calling the C2 basket (EMD-21610) (Fig. 1A). The mini-coat, football, tennis ball, and barrel structures match four of the cage geometries that were recently reported by Morris *et al.* (*14*) from in vitro reconstituted clathrin cages, where they used a library of pregenerated cage geometries to sort their particles into the representative cage structures. This indicates that cellular processes produce discrete clathrin geometries in CCVs that are similar to clathrin cages generated by reconstitution. However, there are a few exceptions; we were not able to unambiguously generate the C1 cage that Morris *et al.* (*14*) observed by refining against pregenerated cage geometries. It is possible that the C1 cages are in our dataset, but they are likely in such low abundance that we cannot unambiguously identify their presence. Similarly, we observed other structures larger than barrel CCVs in our dataset (fig. S4) but could not unambiguously reconstruct them. The largest reconstructed cage (36 triskelia) in our dataset is the smallest among eight cages reported by Cheng *et al.* (*13*) that were purified as intact CCVs. However, this difference is likely due to the fact that we did not enrich for larger CCVs by using density gradients in our purification protocol. Instead, we captured the entirety of the CCV population. We have high confidence that our CCVs are representative of SVs because of the abundance of SV proteins identified by mass spectrometry. We also confirmed the presence of vesicles inside the cages by collecting a tomogram of a field of CCVs on the same grid on which we performed the single-particle analysis. This showed that vesicles ranging in size between 22 and 55 nm were present in all the different CCV sizes (fig. S4). The inside of the vesicle is quite dense, and the membrane appears to be loaded with membrane proteins (fig. S4), which is consistent with the model of Takamori *et al.* (*31*). Therefore, the smaller the CCVs get, the denser it becomes inside the vesicles until the membrane and core have about equal density, and there is a dense spherical core of density even in the smallest CCVs (fig. S4, top row).

The C2 basket CCV that we observed by cryo-EM is unique in two ways: First, it has not been reconstructed before in cryo-EM datasets, and second, its existence was ruled out by a cage geometry prediction model developed by Schein (*22*) and used by Morris *et al.* (*14*) in structure determination. This model uses a so-called head-to-tail exclusion rule to find possible cage geometries given the number of vertices. The rule predicts that cages with too much dihedral angle discrepancy (causing their polygonal faces to become nonplanar) are unlikely to form. According to this model, fullerene-like cages with 30 vertices are not permitted at all. In contrast, our C2 structure consists of 30 vertices, 12 pentagons, five hexagons, and 45 edges. Although this structure violates the head-to-tail exclusion rule, it follows Euler’s polyhedron formula, which governs the geometry of closed polyhedrons. The specific region of this geometry that violates the head-to-tail rule consists of four adjacent pentagons with five mutually shared edges (fig. S5A). Four vertices belonging to two of these pentagons lie at the mathematical points where the rule is violated (fig. S5A, arrows). As a result, excessive torsion is present in the surrounding clathrin chains, which explains the slightly weaker density for the CHC chains highlighted in fig. S5A.

The refined clathrin cages were further subjected to subparticle refinement (*33*) to improve the resolution of the clathrin layer. The asymmetric vertex (surrounded by hexagonal-pentagonal-pentagonal or HPP faces) of the mini-coat was refined to 6.3 Å [Fourier shell correlation (FSC _0.143_)] (fig. S3B) overall, with some areas as high as 4.6 Å (EMD-21611) (Fig. 1B and fig. S3D). The hexagonal (EMD-21616) and pentagonal (EMD-21615) faces refined to 9.1 and 8.3 Å, respectively (fig. S3B). The other cage geometries also produced subparticle reconstructions for the HPP asymmetric vertex with resolution ranging between 8 and 9 Å (fig. S3, B and C). This held true for the asymmetric vertex of the C2 basket structure as well (figs. S3, B and C, and S5B).

### Atomic modeling of the asymmetric vertex structure reveals interactions of the QLMLT motif of the CHC and the stabilizing interactions of the CLC with CHC

**Fig. 2:**
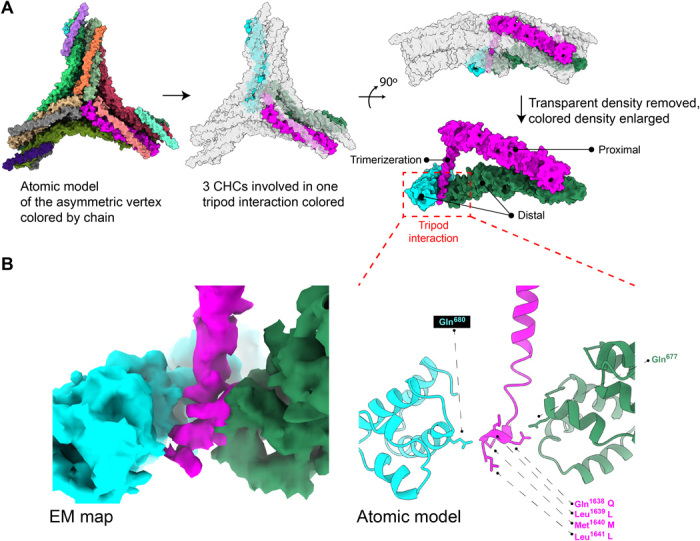
Atomic modeling of the CHC distal leg-tripod interactions. (**A**) The left panel shows the full vertex atomic model. The middle panel shows the same atomic model but highlights the unique interactions of the distal legs from two different CHC chains (green and cyan) with the trimerization domain of a third CHC (magenta). The top right panel is a side view of the model, and the bottom right panel shows only the three interacting CHCs. (**B**) The left panel shows the EM density map for the tripod interaction. The right panel shows the position of the QLML residues of the QLMLT motif extending down from the trimerization helix.

Atomic models were built of the mini-coat HPP asymmetric vertex. The model by Morris *et al.* (*14*) that was built into their consensus C3 vertex [pentagonal-pentagonal-pentagonal (PPP)] was docked into our C1 vertex. In this rigid-body fit, the edge of the vertex that is between two pentagonal faces exhibits an accurate fit, while the two other edges with one hexagonal face do not enclose the model starting at positions close to the knee domain. This discrepancy is consistent with observations that the knee domain changes conformation to make hexagonal and pentagonal faces (*14*), while the proximal domain is stabilized by CLC. Using the asymmetric vertex structure from the mini-coat, a homology model of the bovine CHC and CLC was built and refined to our map [Protein Data Bank (PDB) ID 6WCJ] (Fig. 2A and fig. S6). The CHC C-terminal trimerization domain residues and 15 structurally uncharacterized residues following them were built de novo, including QLML residues of the QLMLT motif (Fig. 2B, right, and fig. S6E) (*34*). The QLMLT motif is critical for Hsc70 binding and clathrin uncoating (*34*). Three trimerization helices of a triskelion (which together form the tripod) sit on top of a triangle made by three distal legs that cross each other pairwise (fig. S7A). We show that each QLML motif extends from the trimerization helix and interacts with two crossing distal legs (fig. S7B).

Another important interaction that happens at the center of the vertex is between the CHC and the CLC. Three C-terminal helices of CLC form a U-shaped domain that lies against CHC residues at the trimerization domain, burying hydrophobic residues along the interface and stabilizing the trimerization domain (fig. S8). This interaction is similar to what was reported by Morris *et al.* (*14*) but with small differences in the atomic model. Our model contains a small helix that is modeled as a loop in the previously published PDB-6SCT (*14*). Our change orients a number of hydrophobic residues to be packed against the trimerization domain (fig. S8).

### Adaptor appendage densities attached to CHC β-propellers are enriched in hexagonal faces

**Fig. 3:**
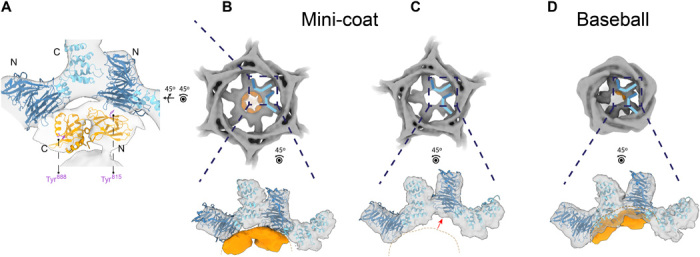
Three-way interaction between β-propeller, ankle, and β2 appendage with their densities and crystal structures shown in dark blue, light blue, and orange, respectively. (**A**) Cross-linking of two β-propellers by a β2 appendage domain. Fitted crystal structures are PDB IDs 1BPO (clathrin) and 1E42 (β2 appendage). (**B**) All hexagonal faces of the mini-coat contain three copies of the β2 appendage density. The dashed boxes indicate magnified insets. The dashed orange arc in the bottom panel roughly outlines the interface between β-propellers and β2 appendage and is used as reference for (C) and (D). (**C**) Pentagonal faces of the mini-coat only have densities for clathrin. Bottom panel shows the movement of the right clathrin leg compared to its position in a hexagonal face as shown in (B). (**D**) Larger cages like the D2 baseball have β2 appendage densities in their pentagonal faces, unlike the mini-coat. However, the position of the β2 appendage in a pentagonal face is different than its position in a hexagonal face [compare (B) and (D)].

Inspection of the CHC β-propeller densities in the mini-coat reconstruction (EMD-21609) revealed that there are three copies of a density consistent with the AP2 β2 appendage domain that reside exclusively in the hexagonal faces and not in the pentagonal faces (Fig. 3). We identify the density as belonging to the AP2 β2 appendage for three reasons. First, the density fits the β2 appendage crystal structure very well (*35*) (Fig. 3A). Second, the AP2 β2 appendage has been shown experimentally to bind and assemble clathrin cages, whereas the other appendage domain–containing adaptors have not (*28*). Last, our CCVs are highly enriched in SVs (fig. S2), and one of the main mechanisms of SV recycling is clathrin mediated and requires AP2. Docking the crystal structure of the β2 appendage in the observed densities aligns residues Y815 of the β-sandwich subdomain and Y888 of the platform subdomain (*16*, *36*) with two neighboring β-propeller domains from two different CHCs (Fig. 3A), effectively cross-linking the two CHCs. For each of the three β2 appendages, one of the two subdomain densities connects down to the body of the adaptor, which allowed us to orient the domains. In the tetrahedral mini-coat, the hexagonal faces lie on threefold axes of symmetry, and three appendages can easily be accommodated by the six β-propellers in the hexagonal face. Since the density value of the appendage domains is less than half of that of clathrin, we estimate that for the mini-coat, there are between one and two AP2 complexes per hexagonal face. In agreement with this, a rough calculation of the total volume of the adaptor layer (volume below clathrin and excluding the vesicle) shows that a total of eight AP2 molecules can exist in a single mini-coat CCV.

The other cage geometries also have an abundance of β2 appendages as well as other extra densities in their hexagonal faces (fig. S9). With the larger geometries, weak appendage densities could also be observed in pentagonal faces that have neighboring hexagonal faces that border each other as discussed in the following sections.

Compared to the reconstituted clathrin cage reconstructions by cryo-EM (*14*, *20*, *37*), our structures have strong densities for the CHC β-propeller domains for all geometries, and they are in contact with the neighboring CHC ankle domain. These contacts are different between the hexagonal and pentagonal faces but consistent across all cage geometries (Fig. 3, B and C, bottom). The differences lie in the region of the β-propeller that is interacting with the ankle and the relative distances and orientations of two neighboring β-propellers. Because of these differences, in pentagonal faces of large cages such as D2 baseball, β2 appendage density is displaced (Fig. 3D) presumably to maintain the same contacts with the β-propellers as those in hexagonal faces (Fig. 3, A and B).

### Pentagonal faces with adaptor appendages have at least two neighboring and consecutive hexagonal faces

**Fig. 4:**
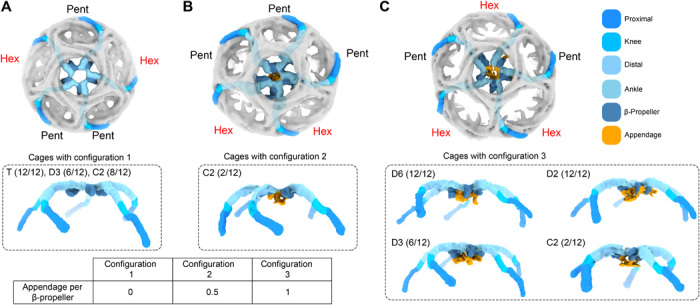
Three different configurations of neighboring faces for a pentagonal face in all geometries and their relation to the population of adaptor appendages in the pentagonal face (colored by domain). Fractions show the number of pentagonal faces in each configuration out of the 12 total pentagonal faces. (**A**) Top: configuration 1. The neighboring faces are indicated with Pent and Hex. The CHC chains with β-propellers in the central pentagonal face are traced using the color code. Bottom: The same CHCs traced in the top panel are shown in a side view with no appendages present. Geometries with configuration 1 are indicated (T, D3, C2). The organization of the top and bottom panels are the same for (B) and (C). (**B**) Configuration 2. This configuration is unique to C2 basket. The bottom panel shows that some β-propellers are engaged with adaptor appendages. (**C**) Configuration 3. The four different geometries indicated in the bottom panel accommodate configuration 3, in which all β-propellers are engaged with adaptor appendages. The table shows a relationship between configuration and approximate fraction of β-propellers in the central pentagonal face of that configuration that are engaged with adaptor appendages.

Unlike hexagonal faces where adaptor appendages are found in abundance in all geometries, appendage densities in pentagonal faces depend on their neighboring faces. In the five resolved cage geometries, there are three possible configurations of neighboring faces for each pentagonal face (Fig. 4, A to C). The first configuration has two non-neighboring hexagonal faces and appears in the mini-coat, football, and basket geometries (Fig. 4A, top). This configuration has no associated adaptor appendages in the pentagonal faces (Fig. 4A, bottom). The mini-coat pentagonal faces are composed entirely of this configuration, which explains why no appendage densities are observed in the pentagonal faces for that geometry. In the football geometry, 6 of the 12 pentagonal faces are in this configuration, and remarkably, none of those faces contain appendage densities. The same is true for the basket geometry. The second configuration is unique to the basket geometry and has two neighboring hexagonal faces that adjoin each other (Fig. 4B, top). In this configuration, two of the β-propellers in the pentagonal face are interacting with adaptor appendages, while the remaining three β-propellers show no appendage densities (Fig. 4B, bottom). The third configuration has three total hexagonal faces, two of which adjoin each other, and this appears in the barrel, football, tennis ball, and basket geometries (Fig. 4C, top). In this configuration, all β-propellers in the pentagonal faces interact with adaptor appendages (Fig. 4C, bottom). This observation clearly shows that the presence of appendages in pentagonal faces depends on having adjacent hexagonal faces, where two of the hexagonal faces adjoin each other (Fig. 4, B and C). This configuration, by necessity, becomes more common with large cages, and accordingly, we observe more appendage densities in pentagonal faces with larger and larger cages (Fig. 4). These results suggest that a cluster of adaptors in adjoining hexagonal faces also engages their neighboring pentagonal β-propellers.

### Subparticle refinement reveals AP2 core density

**Fig. 5:**
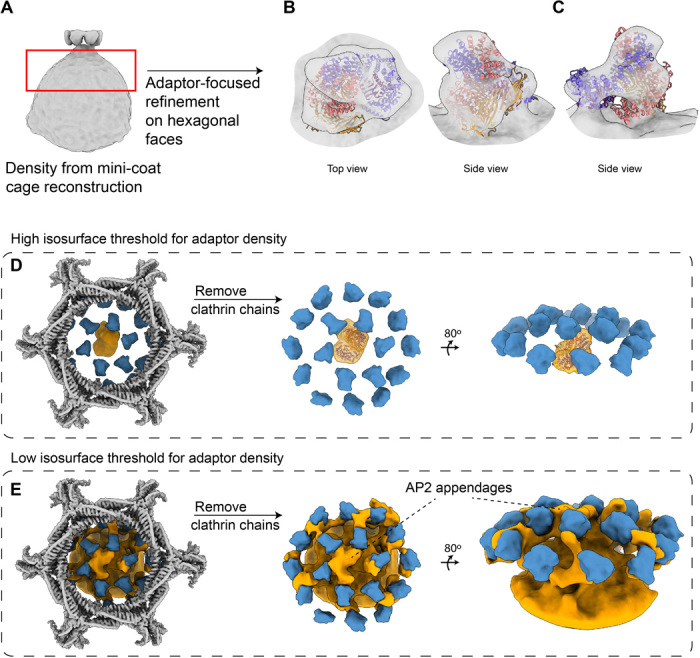
Adaptor-focused refinement revealing AP2 core density and a network of cross-linking densities (clathrin colored by domain). (**A**) β2 appendages of one hexagonal face and averaged-out density from the mini-coat cage refinement. Red box indicates the volume that was further resolved in subparticle alignment. (**B**) Subparticle alignment reveals the AP2 core density. The crystal structure of open AP2 conformation is fitted inside. (**C**) AP2 density at a slightly higher isosurface threshold than (B) to emphasize the two connecting densities. (**D**) Relative positions of clathrin hexagonal face (gray), clathrin β-propellers (blue), and AP2 core densities (orange) are shown. The clathrin hexagonal face density is removed to show the proximity of AP2 to 18 β-propellers. (**E**) Same views of (D) are shown here at a lower isosurface threshold to show the interaction web of β-propellers and the cluster of densities formed around AP2.

Since adaptor densities were present in our CCVs but we were only resolving the appendages, we performed subparticle refinement on the hexagonal faces to resolve the adaptor’s core domain. Hexagonal face subparticles (60,000) were generated from 15,000 mini-coat CCVs. A classification scheme (Fig. 5A and fig. S10) was used that focused on alignment and classification of the density below the CHC layer (Fig. 5, B and C). In this refinement, a spherical focus mask was created that enclosed all the CHC ankles, β-propellers, β2 appendages, and the smeared out spherical AP2 density below them. After multiple rounds of classification and removal of poorly aligning particles, this revealed a class that showed a strong density consistent with the AP2 core domain in the center of the hexagonal faces (Fig. 5, B and C). Although the AP2 density was relatively low resolution, it is distinguished by three features: (i) The core AP2 complex in the open conformation [PDB-2XA7, (*7*)] fits inside the corresponding AP2 density (Fig. 5B); (ii) AP2 density makes two points of contact with the density below (Fig. 5C), corresponding to the reported α2 and μ2 interactions with PI(4,5)P_2_ and cargo peptides, respectively; and (iii) appendage densities connect AP2 to CHC β-propeller domains (Fig. 5E). In addition, at a lower isosurface threshold, multiple densities emerge that engage the β-propellers of neighboring pentagonal faces (Fig. 5E). These observations are consistent with the idea that hexagonal adaptor clusters can engage more than just six hexagonal β-propellers. Our hexagonal face map shows that a total of 18 β-propellers are engaged with a cluster of proteins centered on the AP2 adaptor (Fig. 5, D and E).

## DISCUSSION

Structure determination of natively assembled CCVs has revealed many new insights into the interplay between adaptor binding and clathrin cage assembly. CCVs are highly heterogeneous in size, shape, and composition especially with respect to the membrane and cargo proteins (fig. S2B). Nonetheless, using cryo-EM, we were able to classify the cages into five discrete geometries that represent most of the CCVs that we imaged. Using subparticle reconstruction, we determined the structure of the most abundant vertex, which is asymmetric and formed from the intersection of two pentagonal faces and one hexagonal face. This structure confirmed that the interactions that Morris *et al.* (*14*) observed in their reconstituted clathrin cages were conserved in our natively assembled coats. Consistent with their study, our structure also showed that the CLC C-terminal domain stabilizes the tripod domains that are critical for CHC trimerization. A unique feature of our structure was the C-terminal residues of the CHC trimerization domain that stabilize the pairwise interactions of the three distal legs that pass below the center of the vertex (Fig. 2 and fig. S7). The trimerization helices extend past this region, partially exposing the QLMLT motif that is recognized by Hsc70 (*34*). These observations explain how Hsc70 can compete with the tripod/distal domain interaction to destabilize cages.

One of the most notable features of our structures was the enrichment of adaptor appendage densities in the hexagonal faces of the CCV structures (Fig. 3 and fig. S9), whereas appendage densities existed only in a subpopulation of pentagonal faces (Fig. 4). Adaptor densities only appeared in pentagonal faces when they were bordered by two adjacent hexagonal faces, a situation that occurs more frequently with larger and larger CCVs. This suggested that the adaptor appendage densities that bind pentagonal face β-propellers come from the adaptor cluster of their neighboring hexagonal faces. The pentagonal faces could be classified on the basis of the number and position of their neighboring hexagonal faces regardless of cage size and geometry (Fig. 4). Therefore, regardless of cage geometry, we were able to describe the adaptor content of a CCV solely on the basis of the hexagonal faces.

We also found that individual appendage domains cross-link the two β-propellers of two neighboring CHCs, which, in turn, stabilizes a previously uncharacterized interaction between the β-propeller of one CHC and the ankle of its neighboring CHC (Fig. 3). In this way, the binding of a single AP2 ties together two triskelia and promotes cage assembly. This observation also explains why reconstructions of in vitro reconstituted cages lacking β2 appendages do not show the same ubiquitous β-propeller–ankle interaction that we observe.

Another insight into adaptor-clathrin interactions came from an asymmetric adaptor-focused subparticle refinement on the hexagonal faces. We resolved the AP2 core, extra weaker densities adjacent to the AP2 core, and a whole network of densities that directly link the hexagonal face β-propellers to the 12 CHC β-propeller domains in the neighboring faces. (Fig. 5). We hypothesize that the extra densities that we observe under hexagonal faces and adjacent to the AP2 core density can be assigned to the structured domains of other accessory/adaptor proteins such as AP180 and epsin. This is supported by the studies that have shown that AP2 recruits those proteins to endocytic sites. Furthermore, we hypothesize that the densities linking multiple β-propeller domains can be assigned to the unstructured C-terminal regions of AP180, epsin, and AP2 surrounding their clathrin box motifs. This has been predicted in the literature, since each of these regions has multiple clathrin box motifs that enable them to bind multiple clathrin β-propeller domains, resulting in a cross-linking effect (*29*, *38*).

**Fig. 6:**
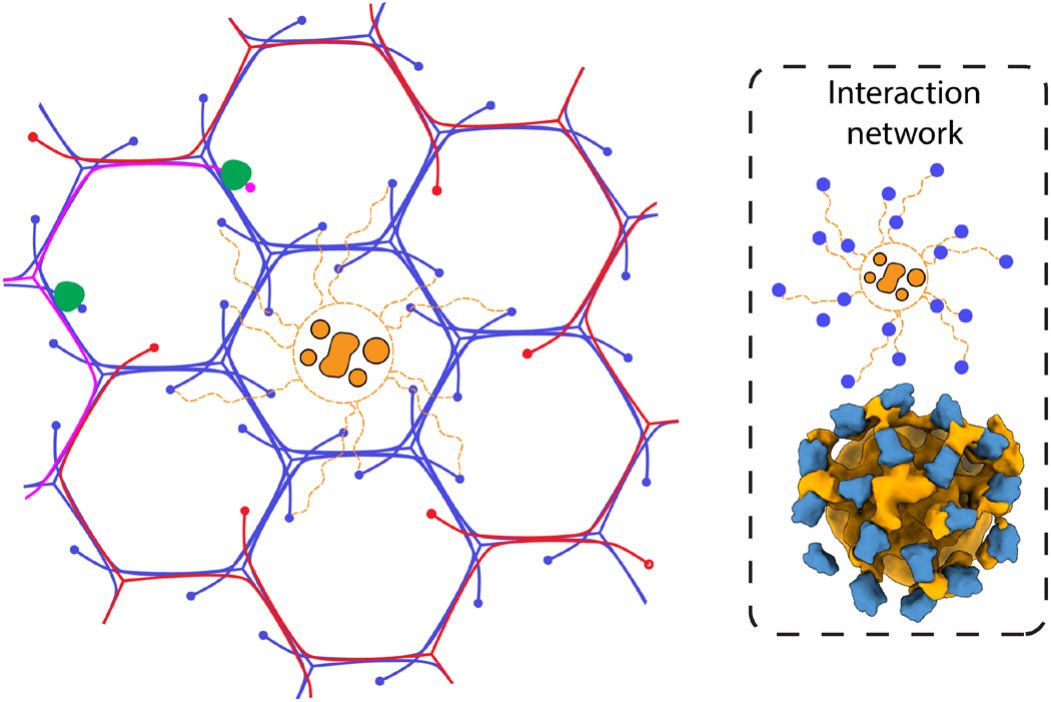
Model for adaptor cluster–mediated organization of pentagonal and hexagonal faces (colored by domain). Auxilin is represented by green blobs sitting on top of β-propellers. Clathrin chains in red and magenta are not interacting with the adaptor cluster and hence can be removed. The orange adaptor cluster and the blue clathrin chains are interconnected with dashed lines. Dashed inset shows the schematic interaction network of β-propellers cross-linked by adaptors and the map on which it was based.

All of these observations have led us to propose a model for how adaptor binding drives the formation of discrete cage geometries. Our data demonstrate that β2 appendages are enriched in hexagonal faces and cross-link the β-propellers of neighboring clathrin triskelia and that there is a cluster of densities surrounding AP2 and connecting the hexagonal β-propellers to the neighboring pentagonal β-propellers. For the formation of a closed cage, there should be a mechanism that explains how pentagonal faces are formed in the two modes of assembly: constant area and constant curvature (*11*, *39*). In the constant curvature model, pentagonal faces would form in membrane regions lacking adaptor clusters to accommodate membrane curvature. For the constant area model, CCVs start out as a flat hexagonal lattice, and triskelia have to be removed by Hsc70/auxilin to convert hexagonal faces to pentagonal ones (*40*–*42*). Our data suggest that the adaptor cluster in the center of hexagonal faces (Fig. 6, orange) ties together the 18 surrounding β-propellers and their associated triskelia (Fig. 6, blue), thus preventing their removal. In this view, these triskelia would remain even if their tripod interactions were disturbed temporarily by Hsc70/auxilin. This means that the only triskelia available for removal would be the distant ones that are not interacting with the adaptor cluster (Fig. 6, red). In the neighboring hexagonal faces, there is only one triskelion per face that is not engaged in any adaptor hub interactions (Fig. 6, magenta and red). If that triskelion was removed from all the six faces by Hsc70/auxilin (Fig. 6, green), then the adaptor cluster would give rise to a hexagon surrounded by six pentagons, thus accommodating a curved membrane. To make larger cages, more hexagonal faces must be maintained via additional adaptor clusters. Thus, in our model, the positioning of the 12 pentagons that are required for the generation of a closed cage is not determined by the geometrical constraints of clathrin but rather by the positioning of adaptors. This central role of adaptors in generating clathrin geometries is supported by our observation of the C2 basket, which should be unlikely to form on the basis of clathrin interactions alone. Our model is consistent with both the constant area and the constant curvature models since it does not assume any initial long-range geometrical order and is solely based on local adaptor clusters. Since the adaptor cluster around AP2 contains curvature-generating proteins such as epsin and AP180 and is centered under hexagonal faces, the clathrin chains that can be removed from the flat lattice (Fig. 6, magenta and red) are at the rim of the curved membrane, where hexagonal faces should be switched to pentagonal faces. In these ways, our model unifies the literature on CCV assembly by describing clusters of adaptors as the central machinery for curvature generation and CHC cage assembly.

### Accession codes

All cryo-EM maps and structures have been deposited in the Electron Microscopy Data Bank and PDB with the following IDs: (i) EMD-21609, full mini-coat reconstruction with β2 appendage densities; (ii) EMD-21608, clathrin-focused mini-coat reconstruction; (iii) PDB ID 6WCJ, EMD-21611, asymmetric vertex of the clathrin mini-coat cage; (iv) EMD-21615, pentagonal face of the mini-coat; (v) EMD-21616, hexagonal face of the mini-coat; (vi) EMD-21610, basket geometry; (vii) EMD-21612, football geometry; (viii) EMD-21613, tennis ball geometry; (ix) EMD-21614, barrel geometry. Additional data are available from authors upon request.

## METHODS

### Purification

All the following steps were done at 4°C. Cow brain (120 g) purchased from Pel-Freez Biologicals was homogenized in MES buffer [100 mM MES, 1 mM EGTA, and 0.5 mM MgCl_2_ (pH 6.7)] and two protease inhibitor cocktail tablets using an industrial blender (final volume of 300 ml) (*43*). The homogenate was centrifuged at 17,000*g* (rotor JA-25.50, 25 min), and the supernatant was transferred and centrifuged at 56,000*g* (rotor 70Ti, 1 hour). The pellet from that run was resuspended in MES buffer and 12.5% (w/v) of each sucrose and Ficoll and then homogenized again using the blender. The homogenate was spun at 43,000*g* (rotor 70Ti, 1 hour) to pellet large microsomes. The supernatant was transferred and spun at 100,000*g* (rotor 70Ti, 2.5hours). The pellet was resuspended in MES buffer and spun at 17,000*g* (rotor JA-25.50, 10 min) to remove any remaining unwanted large lipids. The intact CCVs (two tubes of 7.5 ml) were then spun on an 8% sucrose cushion dissolved in MES buffer made with D_2_O (2.5 ml, added to the bottom of the sample) at 116,000*g* (rotor SW41Ti, 2.5 hours) to separate out similar-sized uncoated vesicles (*43*). The pellet was resuspended in MES buffer at a concentration of 6.5 mg/ml.

### Mass spectrometry

#### *Protein digestion*

Cut gel bands were destained with a wash buffer (50% aqueous acetonitrile with 50 mM ammonium bicarbonate) and cut into ~1-mm gel pieces and dried by incubation with acetonitrile followed by SpeedVac (Thermo Fisher Scientific). Digestion buffer (10% aqueous acetonitrile with 50 mM ammonium bicarbonate) was added to rehydrate the dried gel pieces, which subsequently were reduced with 0.5 mM TCEP [tris(2-carboxyethyl)phosphine; catalog no. C4706, Sigma-Aldrich, St. Louis, MO] and alkylated with 1 mM IAA (iodoacetamide; catalog no. I1149, Sigma-Aldrich, St. Louis, MO). Then, trypsin (catalog no. 90058, Thermo Fisher Scientific, Waltham, MA) was added to a final concentration of 0.005 μg/μl, and the mixture was incubated at 37°C overnight. Digestion was quenched by adding 0.5% formic acid aqueous solution, and the supernatant was collected. The residual gel pieces were dried by incubation with acetonitrile, and the supernatant was collected. Combined supernatant containing tryptic peptides was dried in the SpeedVac.

For the in-solution protein trypsin digestion, 24.5 μl of the digestion buffer (10% aqueous acetonitrile with 50 mM ammonium bicarbonate) was added to 0.5 μl of the crude CCV sample. The mixture was then reduced with 0.5 mM TCEP, alkylated with 1 mM IAA, and digested with trypsin (0.005 μg/μl) at 37°C for 3 hours. The digestion was quenched by adding 0.5% formic acid aqueous solution, and the tryptic peptide mixture was dried in the SpeedVac.

#### *Tryptic peptides separation and detection*

The peptide mixture was redissolved in 0.1% aqueous formic acid and separated by nano–liquid chromatography (nLC) with an EASY-nLC II system (Thermo Fisher Scientific) with a 100-μm–by–2-cm trap column (EASY-Column™ Capillary Columns, catalog no. SC001, Thermo Fisher Scientific) and a 75-μm–by–10-cm C18AQ analytical column (Thermo Fisher Scientific). Mobile-phase compositions are A (0.1% aqueous formic acid) and B (99.9% acetonitrile and 0.1% formic acid). For the crude CCV sample, a linear gradient from 5 to 35% B over 2 hours was performed with a flow rate of 300 nl/min. For gel band samples, the linear gradient was from 5 to 35% B over 1 hour. Eluate was on-line ionized by nano–electrospray ionization with a spray voltage of 2.2 kV and detected by a Hybrid Velos Linear Trap Quadropole (LTQ)–Orbitrap mass spectrometer (Thermo Fisher Scientific). Both nLC and mass spectrometry were controlled by Xcalibur software (Thermo Fisher Scientific). The precursor ions were detected with a mass resolution of 60,000 (at a mass/charge ratio of 800 Da) and an automatic gain control (AGC) of 1.00 × 10^6^ in the Orbitrap. Centroided data-dependent tandem mass spectrometry was carried out on the top 10 most abundant precursor ions with collisional induced dissociation in the LTQ with AGC of 1.00 × 10^4^.

#### *Data analysis*

Acquired Xcalibur.raw files were analyzed by Proteome Discoverer software (version 1.4) with a Sequest HT search engine (Thermo Fisher Scientific) against a *Bos taurus* protein fasta database with dynamic modification of methionine oxidation and static modification of cysteine carbamidomethylation. The search result was further validated and visualized with Scaffold software (version 4.3.4, Proteome Software Inc., Portland, OR).

### Cryo-EM data collection and preliminary processing

Sample (3 μl) was applied to thick C-flat grids (CF-2/2-4C-T-5), blotted (blotting time, 4 s; blotting force, 2), and plunged in liquid ethane using a Vitrobot Mark IV (chamber humidity 100% at 4°C). Movie frames were collected using Leginon for automated data acquisition on a Titan Krios equipped with a K3 direct electron detector at ×33,000 magnification, super-resolution mode with a pixel size of 1.37 Å, a total dose of 40.98 e^−^/Å^2^, and a defocus range of 3 to 5 μm. Eight hundred micrographs were obtained after motion correction and dose weighting using MotionCorr2. Contrast transfer function (CTF) was estimated using CTFFIND v4. CCV particles were picked using a FindEM template picking in Appion, resulting in a total of 80,000 CCV particle images (894-pixel box size). FSC was generated using even and odd masked half maps, and the local resolution for the mini-coat vertex was calculated using Monores.

### Cage structure determination

The pool of 80,000 CCV particle images was 2D-classified using cryoSPARC, leaving 60,000 particles which were then 3D-classified using Relion and cisTEM into five different clathrin cage geometries:

1) Mini-coat [15,000 particles, T symmetry, 28 triskelia, 24 C1 (HPP) vertices, four C3 vertices, four hexagonal faces] (EMD-21608 clathrin-focused, EMD-21609 with β2 appendages).

2) C2 basket (8,320 particles, C2 symmetry, 30 triskelia, 22 C1 HPP vertices, four C3 vertices, four C1 vertices, five hexagonal faces) (EMD-21610).

3) Football (6,300 particles, D3 symmetry, 32 triskelia, 24 C1 HPP vertices, two C3 vertices, six C1 HHP, six hexagonal faces) (EMD-21612).

4) Tennis ball (10,200 particles, D2 symmetry, 36 triskelia, 24 C1 HPP vertices, 12 C1 HHP vertices, eight hexagonal faces) (EMD-21613).

5) D6 barrel (8,280 particles, D6 symmetry, 36 triskelia, 24 C1 HPP vertices, 12 C1 HHP vertices, eight hexagonal faces) (EMD-21614).

The C2 basket geometry was delineated from a pool of football particles that were classified into two classes using cisTEM, with the D3 map as the initial model and no symmetry applied.

As a last step, to prepare cage maps and metadata (Euler angles) for subparticle analysis, the Euler angles were refined in cisTEM with no symmetry applied, and then the maps were reconstructed with symmetry in Relion to enable consistent subtraction of projections from images during the subparticle analysis (refer to the next section) and also to adjust the gray scale of the maps to that of the particle images.

### Subparticle analysis

For each of the five cage geometries, three sets of subparticles were defined: vertices, pentagonal faces, and hexagonal faces. There are 12 pentagonal faces in any of the given cage geometries, but the number of hexagonal faces increases with the diameter of the cage. Vertices are divided to three subcategories. The most abundant vertex geometry in any cage geometry is one made by two pentagonal faces and one hexagonal face (HPP). The other two vertex geometries (PPP and HHP) are either absent in some of the cages or vary in number between different cages.

All the subparticles were extracted using the localized_reconstruction.py script (*33*) implemented in Scipion and adopted to work with Relion 3.0 rather than Relion 1.4. The input parameters for the localized reconstruction script included the following: (i) STAR file of refined particle images of a cage; (ii) a map of the corresponding cage, missing the subparticle of interest, to be projected and subtracted from particle images; (iii) C1 symmetry for both particles and subparticles; (iv) coordinates of the center of the subparticles in CMM format, determined such that the CHC part of the subparticle is always centered in its new subparticle box; (v) a subparticle box size of 600 pixels (unbinned); (vi) *z* axis alignment enabled. The subparticles were extracted from the parent cage particle image. Stacks of both subtracted and nonsubtracted subparticles images were created, both unbinned and binned by 2. The subtracted images were only used for high-resolution refinement of clathrin at vertices. First, subparticles were recentered to eliminate centering errors of the extraction step using cryoSPARC 2D classification and alignment. Then, subparticles were refined in cryoSPARC against an initial model extracted from the corresponding cage map. The only symmetry applied for subparticle refinement was C3 for PPP vertices.

### Heavy chain refinement to high resolution

CryoSPARC maps and metadata were exported and refined further in cisTEM. To achieve a higher resolution, we focused on the asymmetric vertex of the mini-coat (EMD-21611), since the mini-coat CCV reconstruction had the highest resolution among the different geometries. At this point, the images were unbinned, and the super-resolution pixels were being used in refinement. After refinement in cisTEM, vertex map and metadata were exported to Relion for CTF refinement, where per-particle defocus and beam tilt were refined. Afterward, the final map was generated by refinement in cisTEM.

### Adaptor classification and analysis

After refinement of CHC (and CLC) of hexagonal (EMD-21616) and pentagonal (EMD-21615) faces of each cage geometry in cryoSPARC, the faces were classified individually in Relion into two classes. Centered at β-propellers, a spherical mask with a diameter of 196 Å was applied such that the hypothetical space occupied by adaptors below β-propellers and the inner distal and proximal clathrin legs in that face were all weighted by one, and everything outside the spherical mask weighted by zero with a Gaussian falloff at the edge of the mask. In that manner, 10 hexagonal and pentagonal subparticles were classified into 20 classes initially.

Sixty thousand hexagonal face particle images from the mini-coat were first classified into two classes. Each class contained 30,000 particles, and both resolved a similar density that resembled the AP2 core density. Therefore, each of the classes was classified further into two classes. Again, the same density was resolved with slightly more features and in a seemingly different orientation. Two more classification steps revealed the AP2 core density presented in Fig. 5. A similar hierarchical classification scheme was carried out for the pentagonal face of the mini-coat. Unlike the hexagonal face, no adaptor density was resolved for the pentagonal face.

### Model building

An initial model for *B. taurus* CHC and CLC was created from the previously published cryo-EM clathrin model [6SCT (*14*)] from *Sus scrofa*. The sequence for CHC in *B. taurus* is identical to *S. scrofa*. The CLC sequences are 96.5% identical, and the nonidentical *S. scrofa* residues were converted to *B. taurus* ones by mutating them in UCSF Chimera. The homology model for a single leg of the triskelion was rigid-body fit into our asymmetric vertex map. The density for hydrophobic interactions between the heavy chain and the light chain in conjunction of other bulky side chain densities were in good agreement with the model. Structural differences were noted for CLC residues 188 to 228 between our map and the porcine map, so these were remodeled in six-residues steps using Rosetta enumerative sampling (RosettaES) and residues 189 to 199 from the 6SCT model as an initial model. On each step, a larger section of the map was segmented, allowing for the six residues to be modeled into the new density. In addition, compared to the porcine map, we resolved additional CHC tripod residues. Using RosettaES and residues 1596 to 1626 from 6SCT, 15 additional residues were modeled. For the remaining legs of the triskelion, the leg structure that we modeled as described above was rigid-body fit into the corresponding densities. Since our vertex density was not symmetric, only the proximal domains of the model fit perfectly into the density. The atoms lying outside of the map were moved into the density using Rosetta torsion space refinement that generally preserves secondary structure and side chain interactions. After the complete vertex model was generated, it was refined using the Rosetta cryo-EM refinement protocol, resulting in 3000 refined models. The models were sorted by total energy, and the model with the lowest energy was selected for further refinement using phenix.real_space_refine. The PDB ID of the final atomic model is 6WCJ. Molecular graphics images were produced using the UCSF Chimera package from the Computer Graphics Laboratory, University of California, San Francisco (supported by the National Institutes of Health (NIH) P41 RR-01081).

## Supplementary Material

aba8397_SM.pdf

## SUPPLEMENTARY MATERIALS

Supplementary material for this article is available at http://advances.sciencemag.org/cgi/content/full/6/30/eaba8397/DC1

## Appendix

View/request a protocol for this paper from *Bio-protocol*.
