# Supplementary material for: The structures of natively assembled clathrin-coated vesicles
Source: Sci Adv. 2020 Jul 22;6(30):eaba8397. doi: 10.1126/sciadv.aba8397 (PMC7375819; doi:10.1126/sciadv.aba8397)
Supplement: aba8397_SM.pdf [file aba8397_SM.pdf]

Science Advances
AAAS

advances.sciencemag.org/cgi/content/full/6/30/eaba8397/DC1

Supplementary Materials for

The structures of natively assembled clathrin-coated vesicles

Mohammadreza Paraan, Joshua Mendez, Savanna Sharum, Danielle Kurtin, Huan He, Scott M. Stagg*

*Corresponding author. Email: sstagg@fsu.edu

Published 22 July 2020, *Sci. Adv.* **6**, eaba8397 (2020)
DOI: 10.1126/sciadv.aba8397

This PDF file includes:

Figs. S1 to S10

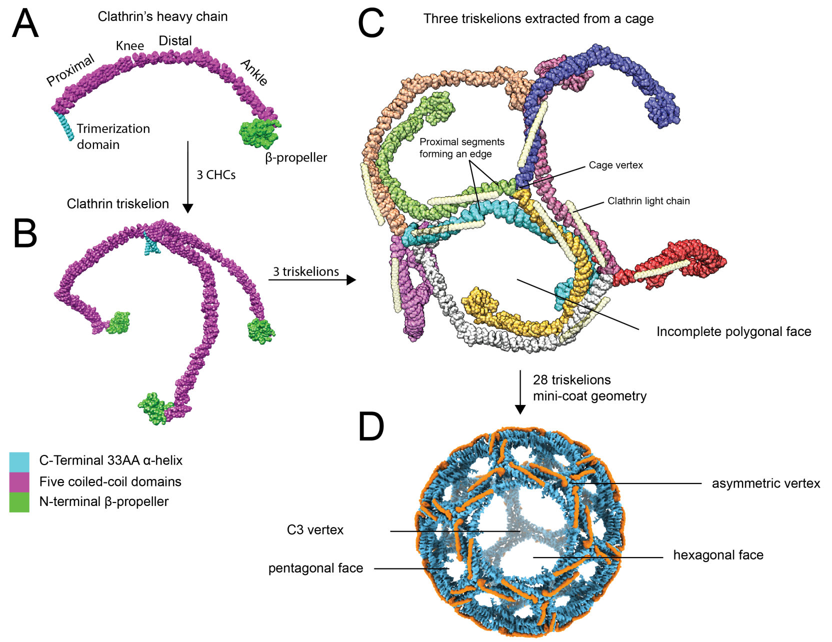

Figure S1. Assembly of clathrin heavy chain at different stages. Color code for panels A and B is the same and it is different from C and D. A) A single leg-shaped clathrin heavy chain is represented with the names of the domains that each engage in specific interactions. B) Three clathrin heavy chains form a triskelion by interacting through the long α-helical trimerization domain. C) Each CHC has a single color, CLC is represented in light yellow. Interactions between different domains are shown for three triskelions. Two anti-parallel proximal legs make the top layer of an edge, and two anti-parallel distal legs make the bottom layer of an edge. Each triskelion constitutes the center of a vertex, while three knee domains pass near the center, and three distal legs pass under the center of the vertex. Light chain's heptad repeat lies against the proximal domain. β-propellers lie inside polygonal faces. D) Cryo-EM map of the mini-coat. CLC is shown in orange and CHC is shown in blue. Different geometrical elements of the structure are highlighted. There are 4 hexagonal faces, 12 pentagonal faces, 24 asymmetric vertices, and 4 C3 vertices in the mini-coat structure.

A

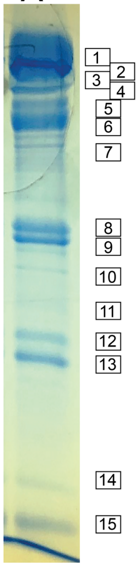

Gel band	Identified Protein	Accession #	Total Spectra
1	Synaptosomal-associated protein, 91kDa homolog	DAA26365.1	69
2	AP2-associated protein kinase 1 isoform X1	XP_015320883.1	62
3	Clathrin heavy chain 1	CLH1_BOVIN	482
4	Sodium/potassium-transporting ATPase subunit alpha-1	AT1A1_BOVIN	49
	AP-3 complex subunit beta-2	NP_001230264.1	20
5	AP-2 subunit alpha-1	NP_001178369.1	94
	Amphiphysin isoform X2	XP_010802730.1	23
6	AP-1 subunit beta-1	AP1B1_BOVIN	118
	AP-2 complex subunit alpha-2	AP2A2_BOVIN	105
	AP-1 subunit beta-1 isoform X1	XP_005218100.1	40
	AP-1 subunit gamma-1 isoform X1	XP_005218546.1	17
7	Epsin-4	EPN4_BOVIN	42
	Epsin-1	NP_001033759.1	18
	AP-3 subunit delta-1	AP3D1_BOVIN	4
8	Tubulin alpha-1D chain	TBA1D_BOVIN	62
9	AP-2 subunit mu	AP2M1_BOVIN	75
	AP-1 subunit mu-1	AP1M1_BOVIN	4
10	AP-3 mu3A subunit	AP3M1_BOVIN	3
11	Synaptophysin	DAA12827.1	6
	Adaptin ear-binding coat-associated protein 1	NECP1_BOVIN	23
12	Syntaxin-1B	STX1B_BOVIN	18
	Syntaxin-1A	NP_001077267.1	5
13	Clathrin light chain B	CLCB_BOVIN	88
14	Ferritin light chain	FRIL_BOVIN	74
15	Ferritin heavy chain	FRIH_BOVIN	151
	AP-2 complex subunit sigma	AP2S1_BOVIN	15
	AP-1 subunit sigma-1A	AP1S1_BOVIN	7
	AP-1 subunit sigma-1B	AP1S2_BOVIN	7
	Synaptobrevin-2	VAMP2_BOVIN	29

B

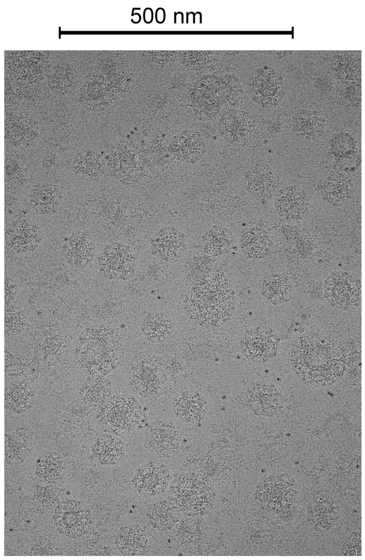

Figure S2. Natively assembled CCVs purified from cow brain. A) On the left, an SDS-PAGE gel lane of the crude sample is shown with bands numbered from high to low molecular weight. In the table, for each gel band, the identity with the highest normalized total spectra count is listed. AP180 is band 1, listed with its gene name, synaptosomal-associated protein (SNAP91). Mass spectrometry of the crude sample in solution (not shown) agrees with the gel-band analysis. B) An exemplar micrograph of natively assembled CCVs at 33k magnification. Scale bar is 500 nm. Dark spots are ferritin molecules that co-purified in the centrifugation process.

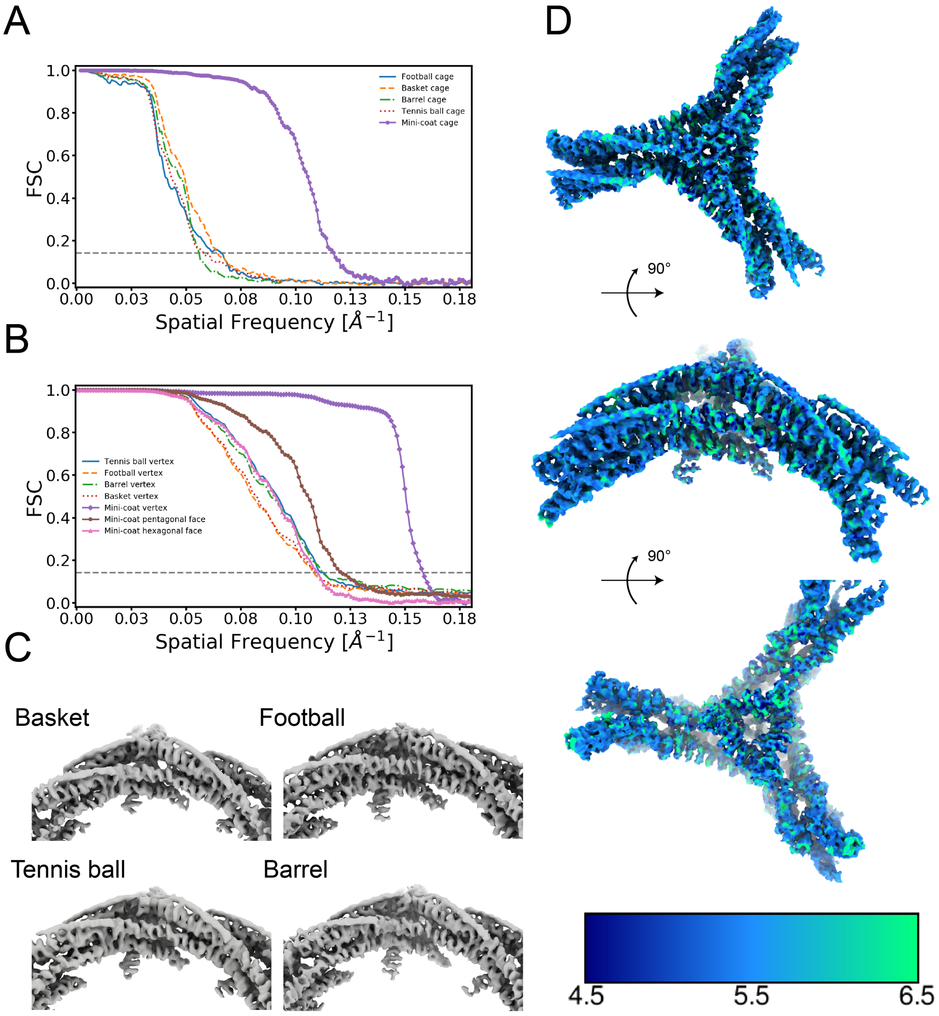

Figure S3. Single particle analysis of cages and subparticles. A) $FSC_{0.143}$ values for cage reconstructions are: football-15.1 Å, basket - 15.6 Å, barrel - 17.9 Å, tennis ball - 17.2 Å, and mini-coat - 8.5 Å. B) $FSC_{0.143}$ values for subparticle reconstructions are: tennis ball vertex - 8.97 Å, football vertex - 9.24 Å, barrel vertex - 8.9 Å, basket vertex - 9.13 Å, mini-coat vertex - 6.3 Å, mini-coat hexagonal face - 9.1 Å, mini-coat pentagonal face - 8.27 Å. C) asymmetric vertex reconstructions. D) local resolution map for the mini-coat vertex generated by monores. The indicated resolutions are color-coded on the map.

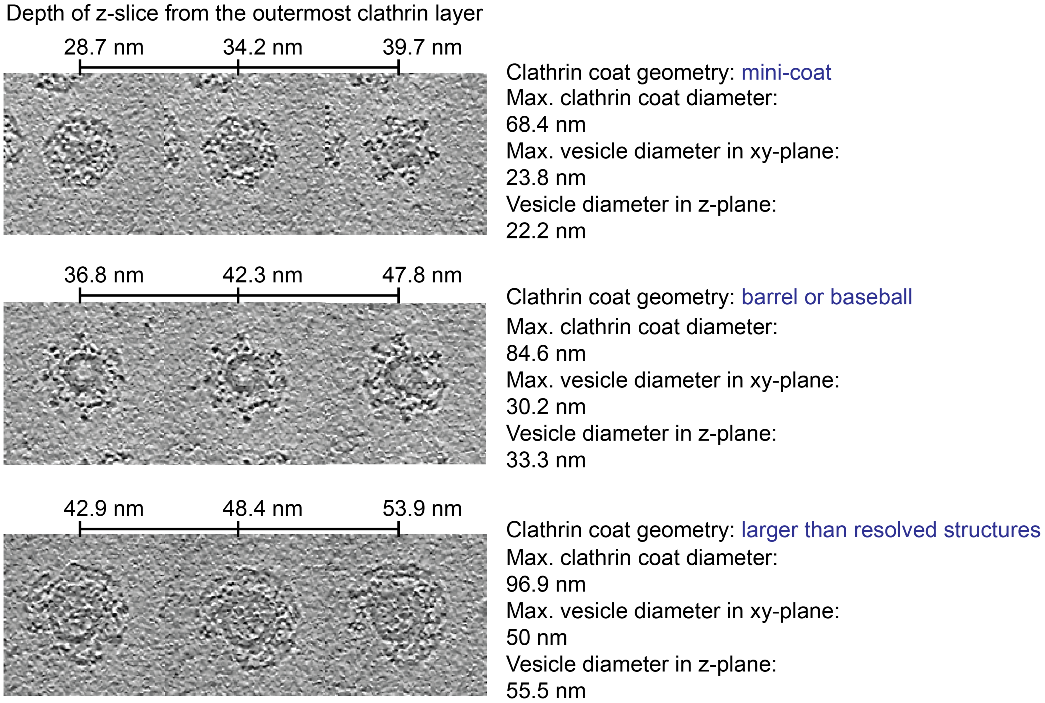

Figure S4. Tomogram slices of three different-sized CCVs show a dense vesicle for the mini-coat and large vesicles with visible bilayers for larger CCVs. Each row contains three slices of the same CCV particle. The CCV in the first row is identified as the mini-coat based on its architecture. The CCV in the second row has dimensions close to that of D2 baseball or D6 barrel. The CCV in the third row is a large under-populated CCV that was not reconstructed in the single particle analysis and hence its geometry is unknown.

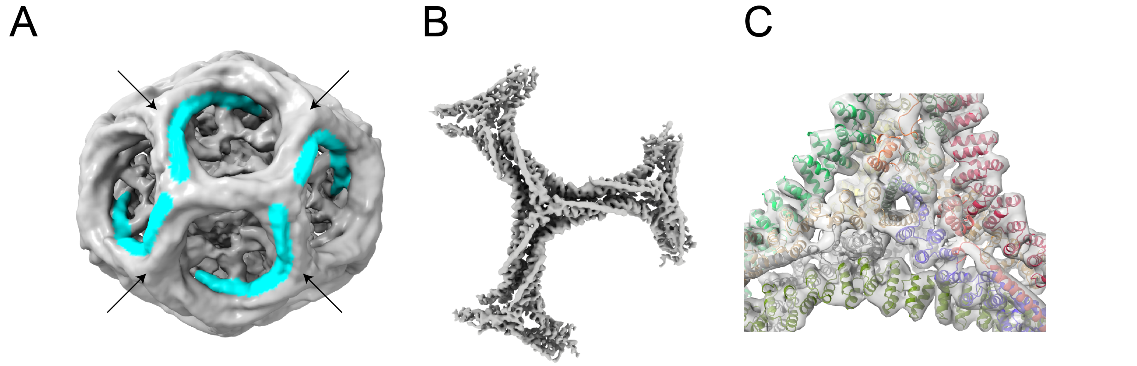

Figure S5. Novel C2 basket geometry with 30 triskelions. A) Arrows show vertices where the head-to-tail exclusion rule is violated. The CHCs highlighted in cyan have a weaker density compared to the rest of the structure. B) Subparticle refinement of the asymmetric HPP vertex of the C2 basket at 9.4 Å. C) Fitting of the generated atomic model for the asymmetric vertex of mini-coat into the asymmetric vertex of the C2 basket (transparent gray) shows no deviation from standard CHC conformations.

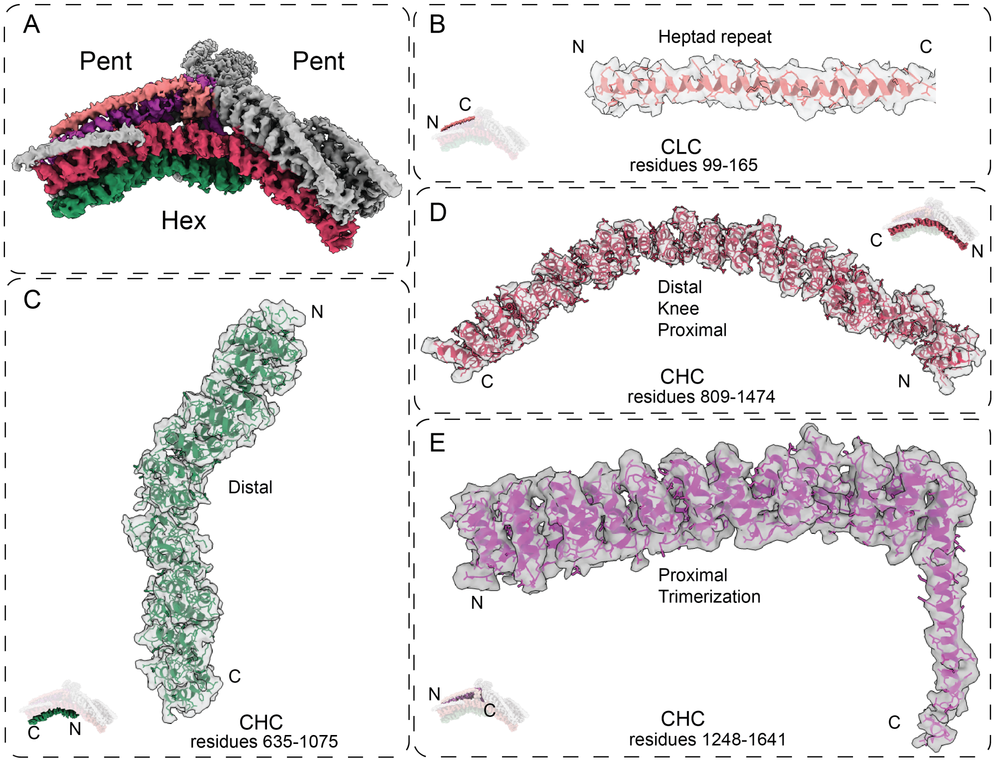

Figure S6. Summary of the unique regions of the refined atomic model from the hexagonal face side of the vertex (colored by chain). N- and C-termini are labeled. Small inset in each panel show where the chain fits with respect to the map in A. B) Map and atomic model of the heptad repeat of the light chain. C) Map and model for CHC residues 635-1075. D) Map and model for CHC residues 809-1474. E) Map and model for CHC residues 1248-1641.

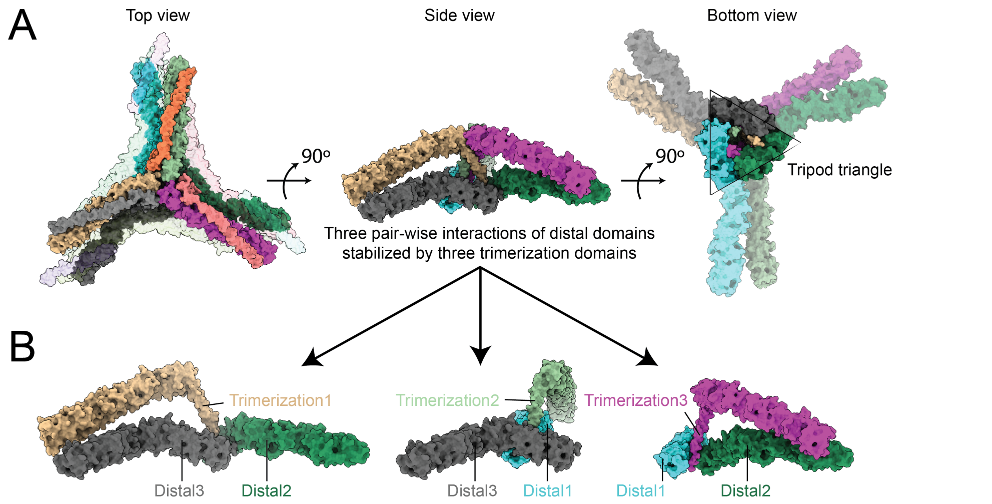

Figure S7. Interactions of the six distinct CHCs comprising the tripod interactions (colored by chain). Transparent densities in the left panel of A are clathrin chains that are not involved in the tripod interactions and are removed in the rest of the panels as well as the light chains. The triangle shape made by tripod interactions is highlighted in the right panel of A. In B, each of the three tripod interactions are isolated.

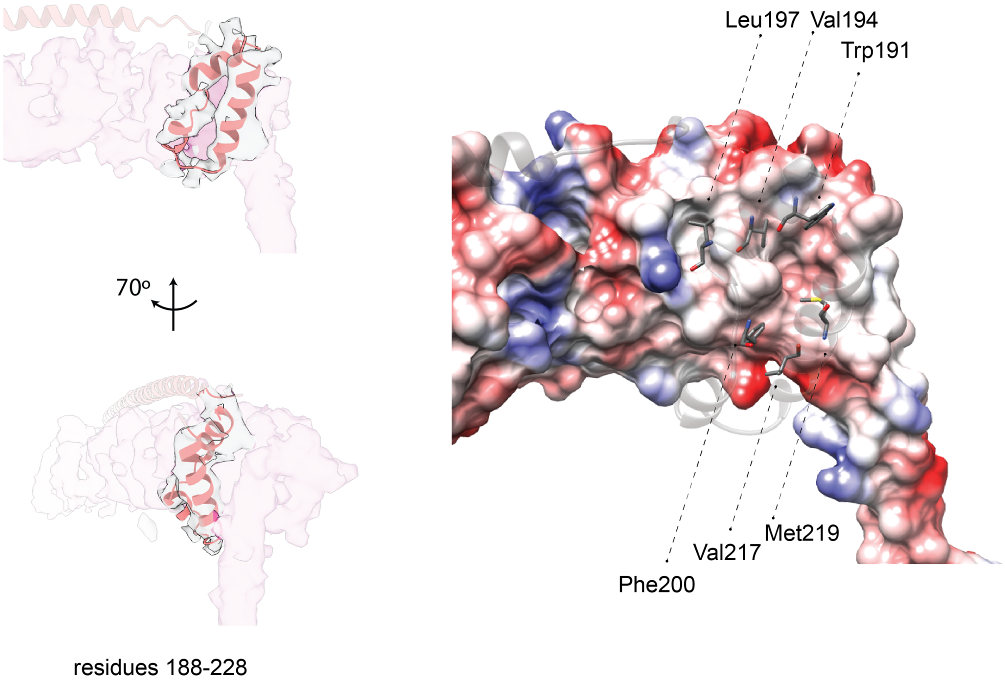

Figure S8. Atomic model of the C-terminal U-shaped domain of CLC and the hydrophobic residues buried against CHC. The left panels show the atomic model fitted in the map in two views. The right panel has the same view as the top left panel. In the right panel, CHC is shown in molecular surface representation with white showing hydrophobic residues and CLC is shown in transparent ribbon representation. The hydrophobic residues of CLC that lie against the CHC trimerization domain are represented as sticks and are labeled.

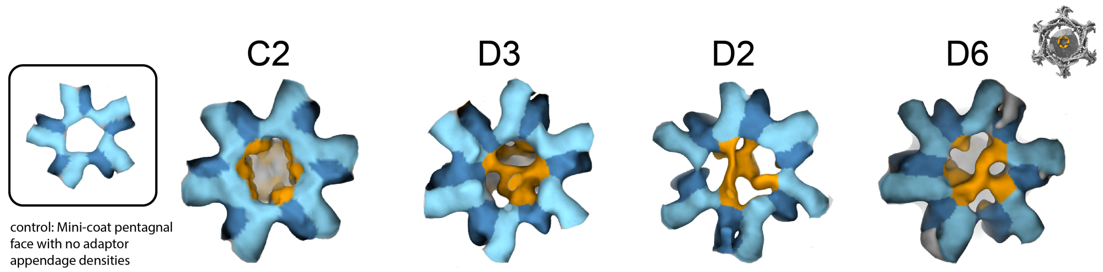

Figure S9. Adaptor appendage densities in hexagonal faces of all cage geometries. At the top right corner, the orientation of all faces is indicated with the hexagonal face of the mini-coat in gray and its β2-appendage in orange. The inset at the left shows a pentagonal face with no adaptor appendages for comparison. The color code is the same as that in Figure 4. β-propellers are in dark blue, the ankle domain is in light blue, and adaptor appendages are in orange. C2 basket, D3 football, D2 tennis ball, and D6 barrel hexagonal faces are shown from left to right, respectively.

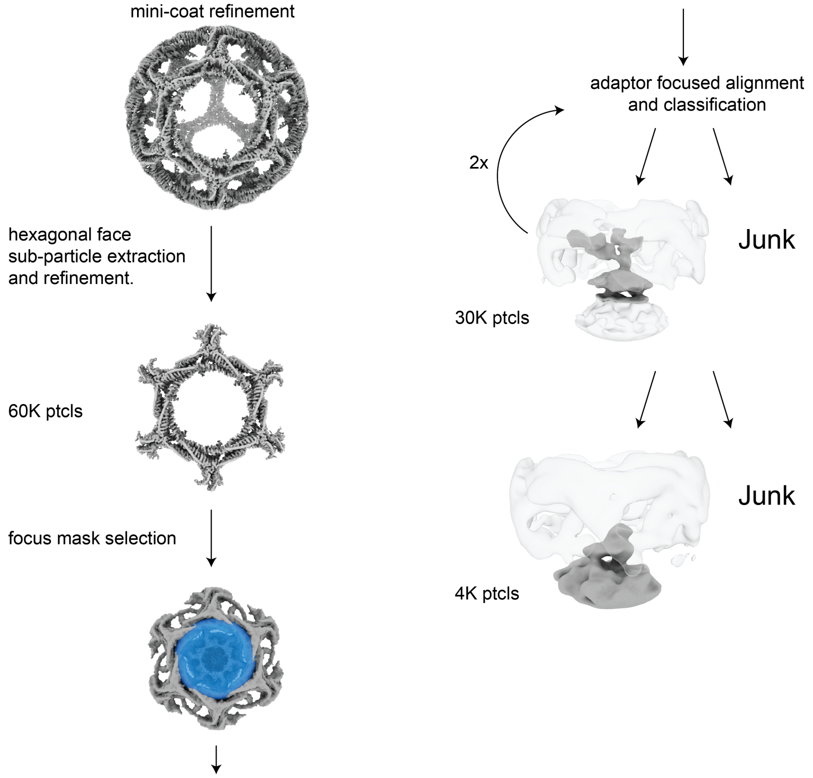

Figure S10. Schematic of adaptor-focused refinement and classification strategy. The first step was a single particle refinement of whole mini-coat CCVs. With the Euler angles of cages and the position of the hexagonal sub-particles known, each face was re-extracted as a new particle image to generate a new sub-particle stack. These were first refined as whole subparticles without applied symmetry, and then aligned/classified using a mask that covers their adaptor layer (transparent blue). The classification scheme was repeated for four rounds where in each round the particles were split between two classes and the poorly aligning particles were eliminated from the next round of classification resulting in a final map where AP2 core density was resolved.
